# Intrahepatic Expression of Fatty Acid Translocase CD36 Is Increased in Obstructive Sleep Apnea

**DOI:** 10.3389/fmed.2020.00450

**Published:** 2020-08-11

**Authors:** Esther Rey, Elvira del Pozo-Maroto, Patricia Marañón, Brittany Beeler, Yaiza García-García, Pedro Landete, Stephania C. Isaza, Ramón Farré, Carmelo García-Monzón, Isaac Almendros, Águeda González-Rodríguez

**Affiliations:** ^1^Research Unit, Hospital Universitario Santa Cristina, Instituto de Investigación Sanitaria Hospital Universitario de La Princesa, CIBERehd, Madrid, Spain; ^2^Respiratory Medicine Department, Hospital Universitario de La Princesa, Instituto de Investigación Sanitaria Princesa Hospital Universitario de La Princesa, Madrid, Spain; ^3^Unitat de Biofísica i Bioenginyeria, Facultat de Medicina i Ciències de la Salut, Universitat de Barcelona, CIBERES, IDIBAPS, Barcelona, Spain

**Keywords:** obstructive sleep apnea, intermittent hypoxia, CD36, steatosis, NAFLD

## Abstract

Nocturnal intermittent hypoxia (IH) featuring obstructive sleep apnea (OSA) dysregulates hepatic lipid metabolism and might contribute to the development of non-alcoholic fatty liver disease (NAFLD) observed in OSA patients. However, further research is required to better understanding the molecular mechanisms underlying IH-induced hepatic lipid accumulation. Therefore, the aim of the present study was to determine the effects of OSA on hepatic CD36 expression and the impact of IH by using a mouse model of OSA. Histological analysis, lipid content and CD36 expression were assessed in livers from subjects who underwent liver biopsy and polygraphic study during sleep, and in livers from mice submitted to chronic IH mimicking OSA. Among those who presented OSA features, NAFLD were significantly more frequent than in control subjects with normal respiratory function (77.8 vs. 36.4%, respectively), and showed more severe liver disease. Interestingly, CD36 expression was significantly overexpressed within the liver of OSA patients with respect to controls, and a significant positive correlation was observed between hepatic levels of CD36 and the values of two well-known respiratory parameters that characterized OSA: apnea/hypopnea index (AHI) and oxygen desaturation index (ODI). Moreover, hepatic lipid accumulation as well as induction of hepatic lipogenic genes, and CD36 mRNA and protein expression were significantly higher in livers from mice exposed to IH conditions for 8 weeks than in their corresponding littermates. This study provides novel evidence that IH featuring OSA could contribute to NAFLD setup partly by upregulating hepatic CD36 expression.

## Introduction

Non-alcoholic fatty liver disease (NAFLD) is characterized by metabolic dysfunction and accumulation of lipid deposits in the livers of patients in whom alcohol abuse is not the causal agent of disease onset (1). NAFLD encompasses a wide range of histologic findings from simple steatosis to non-alcoholic steatohepatitis (NASH) with fibrosis and, ultimately, liver cirrhosis, and hepatocellular carcinoma (2). The number of individuals affected by some clinical form of this chronic liver disease is steadily increasing because NAFLD is highly associated with obesity and type 2 diabetes, being considered the hepatic manifestation of the metabolic syndrome (3).

It is well-known that the liver maintains bodily lipid homeostasis by regulating hepatic free fatty acid (FFA) uptake, lipid synthesis, lipid oxidation, and lipid export; however, an imbalance between these metabolic pathways can lead to an excessive lipid accumulation within the liver (4), being an increased *de novo* lipogenesis and largely an enhanced uptake of FFAs released from insulin resistant-adipocytes the main sources of these lipid accumulates (5). The uptake of FFAs into hepatocytes is mainly dependent on the fatty acid translocase CD36 which, under physiological conditions, is weakly expressed in the liver and its expression increases by a number of different stimuli, such as insulin and lipid metabolites, facilitating the process of FFA uptake (6). Some experimental studies have demonstrated that CD36 plays an important role in NAFLD setup in rodents (7, 8) and, reinforcing this notion, it has been observed that fatty liver attenuates in mice fed high fat diet (HFD) upon either systemic or hepatocyte-specific deletion of CD36 (9, 10). Moreover, a growing clinical evidence suggests that this FFA transporter could play a relevant role in NAFLD pathogenesis in humans as well. In particular, Greco et al. showed that hepatic CD36 mRNA levels correlated with liver fat content in morbidly obese patients (11). In addition, different clinical studies have convincingly shown that the amount of both CD36 mRNA and protein was higher in the livers of biopsy-proven NAFLD patients than in subjects with histologically normal liver (12–14).

An increasing number of clinical studies point out to a potential link between obstructive sleep apnea (OSA), a respiratory disorder featured by nocturnal intermittent hypoxia (IH) and sleep fragmentation, and NAFLD (15–18). To highlight, both OSA and NAFLD are especially prevalent among obese individuals and, more interestingly, the severity of nocturnal IH positively correlates with histological features of NASH in OSA patients (19). Although the underlying molecular mechanisms are not fully understood, it has been reported that IH exacerbated fatty liver in obese mice by inducing hepatic lipid biosynthesis (20) likely due to the upregulation of the HIF1α/SREBP1c signaling pathway (21), and that promoted liver inflammation and fibrosis in mice fed with a HFD (22). However, further research is required to unveil the pathophysiological interplays between IH and lipid accumulation. In that regard, whether IH is able to regulate CD36 gene expression in hepatocytes still remains to be elucidated.

Therefore, the primary objective of this study was to determine the impact of IH on CD36 expression as well as on lipid content in livers from OSA patients with biopsy-proven NAFLD and in livers from mice exposed to IH.

## Materials and Methods

### Patients

This study was performed in agreement with the Declaration of Helsinki, and with local and national laws. The Human Ethics Committee of the Hospital Universitario Santa Cristina (HUSC, Madrid, Spain) approved all procedures (PI-688A). This cross-sectional study included 20 patients with gallstones to whom a programmed laparoscopic cholecystectomy was performed in the HUSC. All participants gave a written consent for a perioperative liver biopsy and a postoperative respiratory polygraphy as part of an experimental protocol designed to evaluate the relationship between sleep disturbances and liver disease. All subjects included drank <20 g/day of alcohol, had no previous respiratory disorders, were not having potentially hepatotoxic drugs, had no analytical evidence of iron overload, and were seronegative for autoantibodies, for hepatitis B virus, hepatitis C virus, and human immunodeficiency virus.

### Sleep Study

The polygraphic studies were performed at night in the Sleep Laboratory of the HUSC (Madrid, Spain). For interpretation, the recommendations of the American Academy of Sleep Medicine (AASM) for the diagnosis of OSA were followed. The apnea/hypopnea index (AHI) was used as diagnostic criteria for severity of OSA: AHI <5, no OSA; AHI 5–14, mild OSA; AHI 15–29, moderate OSA; AHI ≥30, severe OSA. In addition, nocturnal hypoxemia parameters including oxygen desaturation index (ODI), cumulative sleep time percentage with oxyhemoglobin saturation (SpO_2_) <90% (Tc90) and minimum SpO_2_ were analyzed.

### Animal Care and Intermittent Hypoxia Protocol

Twelve-weeks-old C57BL/6J mice were purchased from Charles River Laboratories (Saint Germain sur L'Arbresle, France) and divided into two groups of 10 mice. The control mice (C mice) were placed in conditions of normoxia while the experimental group (IH mice) was subjected to IH conditions. Every minute, IH mice received air containing an oxygen fraction of 5% for 20 s, followed by 40 s of room air, during 6 h per day, 5 days a week for a total of 8 weeks. Control mice were only exposed to room air (23). At the end of the experiment, mice were anesthetized, sacrificed and livers were harvested. All experimental procedures were approved by the Ethical Committee of the University of Barcelona (174/18−10268).

### Histopathology Assessment

Liver sections (5 μm) were embedded in paraffin and cut using a Microm microtome (Midland, ON, Canada). After cutting, sections were stained with hematoxylin (1.09235.0500, PanReac AppliChem, Barcelona, Spain) and eosin (71211, Thermo Fisher Scientific, Inc., Madrid, Spain) and with Masson's Trichrome Solution (Masson Trichome Kit with Aniline Blue 04-010802, Milan, Italy). Once stained, the severity of steatosis was quantified by a single-blind hepatopathologist. Specifically, Kleiner's histological scoring system was employed to evaluate the degree of steatosis, lobular inflammation, hepatocellular ballooning, and the stage of fibrosis (24). The following percentages of steatotic hepatocytes were used in the histological assessment: 0–5% hepatocytes, grade 0; 5–33%, grade 1; 33–66%, grade 2; and >66%, grade 3. Histologic diagnosis of liver biopsies was classified into two groups: simple steatosis without hepatocellular ballooning nor lobular inflammation, also termed NAFL, and NASH. Minimal criteria for NASH included the combined presence of grade 1 steatosis, lobular inflammation and hepatocellular ballooning with or without fibrosis. NAFLD activity score was also calculated for each liver biopsy (24). To this end, three different lobular areas were analyzed in each sample. Representative images were taken using a Nikon Eclipse E400 optical microscope (Nikon, Tokyo, Japan) and the NIS Elements Imaging Software (Melville, NY, USA).

### Assessment of Lipid Accumulation

Liver tissue was embedded in Tissue-Tek® O.C.T.™ Compound (Sakura Finetek Europe, Netherlands). Sections (10 μm) were then cut using a Leica CM1510S cryostat (Leica Microsistemas S.L.U, Barcelona, Spain), stained using an Oil Red O biological stain (Sigma-Aldrich, St. Louis, MO, USA) working solution (60% ORO/isopropanol w:v), and counterstained with hematoxylin (1.09235.0500, PanReac AppliChem). Three different lobular areas were analyzed in each sample and photographed using a Nikon Eclipse E400 optical microscope (Nikon) and the NIS Elements Imaging Software (Melville). Intensity of red stain was quantified using ImageJ Biological Image Analysis (NIH) and reported as the average value in arbitrary units (a.u.).

### Quantitative Analysis of Hepatic Triglycerides

Triglycerides (TGs) were extracted as described previously (25). Briefly, liver biopsy samples (15–20 μg) were homogenized in distilled water. Chloroform (−20°C) and methanol (−20°C) were added to each sample. Samples were centrifuged and the triglyceride-containing layer was collected. Once purified, TGs were suspended in isopropanol and analyzed using a colorimetric kit (SpinReact, Girona, Spain). Absorbance values were obtained using a Dynex Spectra MR Microplate spectrophotometer/computer software (Chantilly, VA, USA) and graphically expressed as mg/dl.

### Protein Extraction and Western Blot Analysis

Liver biopsy samples (15–20 μg) were homogenized in an extraction buffer containing the following: 10 mM ethylene-diamine-tetraacetic acid (EDTA), 50 mM Hepes, 50 mM sodium pyrophosphate, 0.1 mM NaF, 10 mM Na_3_VO_4_, 1% Triton X-100 and protease inhibitors. Protein extracts were stored at −80°C after centrifugation. A small aliquot of sample was used for protein quantification (Bradford method). The samples were then prepared to be loaded into 8% SDS-PAGE gels. After running, proteins were further transferred to Inmunoblot nitrocellulose membranes (BioRad Inc., Madrid, Spain), blocked with 5% non-fat dry milk and incubated overnight with primary antibodies: CD36/SR-B3 (1:1000, NB400-144, Novus Biotechne, Abingdon, United Kingdom) and anti-βactin (1:5000, A-5441, Sigma Aldrich). Then, the corresponding secondary antibodies were added (Santa Cruz Biotechnology Inc., Heidelberg, Germany). Using the Bio-Rad Clarity™ Western ECL Substrate (BioRad Inc.), the immunoreactive bands were visualized by the ImageQuant LASD 4000 digital imaging system (GE Healthcare Europe, Barcelona, Spain). Densitometric analysis of the band was performed using ImageJ Biological Image Analysis (NIH), normalized against the loading control (βactin), and graphically expressed as fold change relative to control condition (1).

### Quantitative Real-Time PCR (RT-qPCR)

RNA was extracted from liver samples using the TRIzol® reagent (Vitro, Sevilla, Spain). Samples were then reverse transcribed using the Reverse Transcription System kit (Promega Inc., Madison, WI, USA). A BioRad T100™ Thermal Cycler was used to carry out the reverse transcription. Quantitative real-time polymerase chain reaction (RT-qPCR) was performed to assess gene expression using a StepOnePlus™ Real Time PCR System Sequence Detector (Thermo Fisher Scientific Inc.). Samples were prepared using a SYBER Green qPCR Kit (Promega Inc.) and d(N)6 random primers were purchased from Metabion (Planegg, Germany). Primer sequences are detailed in Supplementary Table 1. Each sample was run in duplicated, normalized in comparison to *36B4* gene expression, and graphically expressed as fold change relative to control condition (1).

### CD36 Immunohistochemistry

Paraffin-embedded liver biopsy sections (5 μm) were deparaffinized and rehydrated. Sections were then placed in antigen retrieval buffer (10 mM sodium citrate, pH 6–7), boiled for 20 min at 95°C and incubated with a blocking solution for 1 h before being immunostained with the CD36 antibody (1:200, NB400-144, Novus) for 16 h in a moisture chamber. The EnVision™ FLEX Mini Kit, High pH (Link) (Agilent, Santa Clara, CA, USA) was used for visualization according to the manufacturer's instructions. Three different lobular areas were analyzed in each sample and images were captured using a Nikon Eclipse E400 optical microscope (Nikon) and the NIS Elements Imaging Software (Melville). Intensity of CD36 stain was quantified using the FIJI software (NIH) and reported as the average value in arbitrary units (a.u.).

### Statistical Analysis

Categorical variables were presented as percentage and were compared by the Pearson χ^2^ test. Continuous data were shown as standard deviation (*SD*) or standard error of mean (SEM), and were compared using the unpaired *t*-test or Mann-Whitney *U*-test, as indicated. The Spearman's *r*-test was used to evaluate correlations. All statistical analyses were performed using the GraphPad Prism 6 software (GraphPad Software Inc., San Diego, CA, USA) and SPSS statistical software version 24.0 (IBM SPSS Statistics, Armonk, NY), with a *p* < 0.05 considered statistically significant.

## Results

### Characteristics of the Study Patients

Twenty patients undergoing programmed cholecystectomy had both a liver biopsy and a sleep study. Overall, the mean age of the study population was 46.5 years, 14 (70%) were female and 9 (45%) had a diagnosis of OSA by polygraphy (AHI > 5). Patient characteristics from the cohort included in this study are presented in Table 1.

**Table 1:** Demographic, metabolic, biochemical, respiratory, and histological characteristics of the study population.

**Feature**	**Patients without OSA (*n* = 11)**	**Patients with OSA (*n* = 9)**	***p-*value**
Age (years)	39.9 ± 9.6	54.6 ± 10.6	0.004
BMI (kg/m^2^)	26.5 ± 6.7	28.6 ± 5.1	0.201
Waist circunference (cm)	91.8 ± 14.4	105.5 ± 11.6	0.034
Glucose (mg/dL)	92.2 ± 9.3	96.2 ± 11.9	0.359
Insulin (μU/L)	7.2 ± 2.9	9.2 ± 4.5	0.245
HOMA-IR	1.6 ± 0.7	2.1 ± 1	0.192
Triglycerides (mg/dL)	111.2 ± 54.2	136.4 ± 68.9	0.467
Total Cholesterol (mg/dL)	207 ± 32.3	210 ± 29.5	0.832
HDL-cholesterol (mg/dL)	53.5 ± 12.8	46.9 ± 9.5	0.212
ALT (IU/L)	24.6 ± 17.4	27.2 ± 19.3	0.489
AST (IU/L)	22.1 ± 9.3	21.3 ± 5.6	0.808
γGT (IU/L)	33.4 ± 24.4	28.4 ± 14.5	0.601
Alkaline phosphatase (IU/L)	70.3 ± 22.7	61 ± 13.6	0.359
Bilirubin (mg/dL)	0.7 ± 0.36	0.63 ± 0.32	0.780
Average oxygen saturation (%)	94 ± 1.7	93.3 ± 1.4	0.225
Minimum oxygen saturation (%)	88.2 ± 4.2	83.7 ± 5.9	0.073
AHI (events/hour)	0.9 ± 1	10 ± 9.2	0.002
ODI (events/hour)	0.6± 0.6	9.2 ± 9.5	<0.001
Tc90 (%)	7.5 ± 16.9	6 ± 8.8	0.053
**NAS Score (%)**			
0		22.2%	
1	18.2%	22.2%	
2	18.2%	22.2%	
3		11.2%	
4		22.2%	
**Steatosis (%)**			
Grade 0	63.6%	22.2%	
Grade 1	27.3%	22.2%	
Grade 2	9.1%	44.5%	
Grade 3		11.1%	
**Lobular Inflammation (%)**			
Grade 0	91%	77.8%	
Grade 1	9%	22.2%	
Grade 2			
**Ballooning (%)**			
Grade 0	100%	77.8%	
Grade 1		22.2%	
Grade 2			
**Fibrosis (%)**			
Stage 0	100%	100%	

Data are shown as mean ± standard deviation.

*OSA, obstructive sleep apnea; BMI, body mass index; HOMA-IR, homeostatic model assessment-insulin resistance; HDL, high density lipoprotein; ALT, alanine aminotransferase; AST, aspartate aminotransferase; γGT, gamma-glutamiltranspeptidase; AHI, apnea-hipopnea index; ODI, oxygen desatutaion index; Tc90, sleep time with oxygen saturation <90%*.

OSA patients were significantly older than those without OSA (*p* = 0.004) and women predominated in both groups. In order to evaluate the presence of obesity in the entire study population, body mass index (BMI) was calculated and waist circumference was measured in each participant. Regarding BMI, the study population showed an overweight status and no significant differences were found among the different patient groups studied (*p* = 0.201), whereas waist perimeter was significantly higher in patients with OSA than in those without (*p* = 0.034).

As expected, OSA patients had a significantly higher rate of oxygen desaturation per hour of sleep (ODI) and percentage of sleep time with oxygen saturations lower than 90% (Tc90) with respect to patients without OSA (Table 1).

Regarding metabolic parameters, basal glucose levels did not significantly differ between groups, while insulin levels and the degree of insulin resistance assessed by HOMA-IR index were higher in OSA patients than in those without OSA, but these differences were not statistically significant (Table 1).

**Figure 1:**
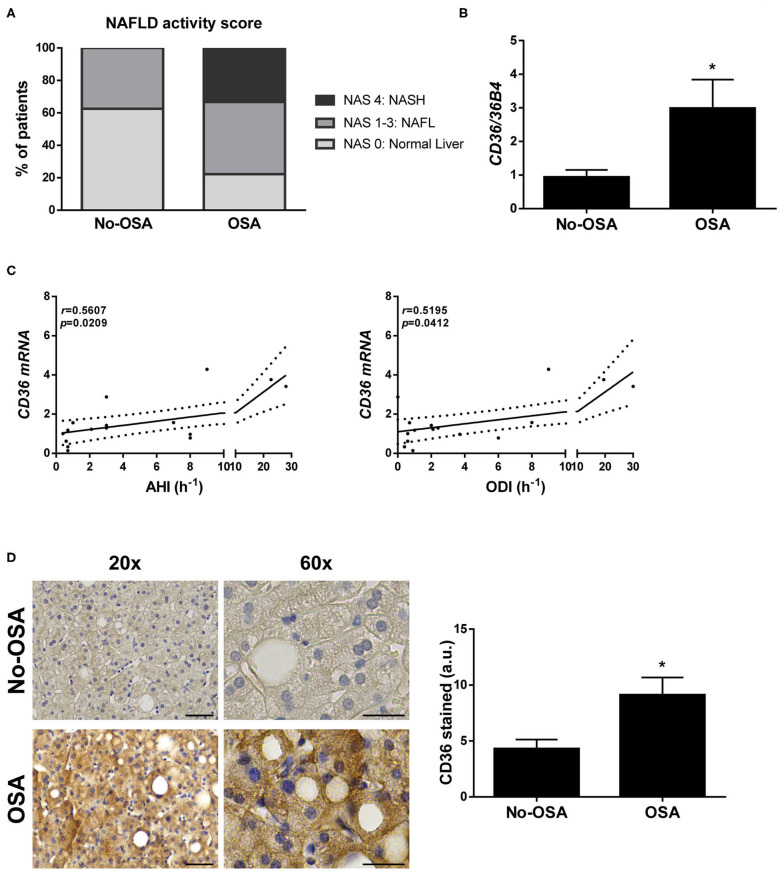
Prevalence of NAFLD and hepatic CD36 expression is higher in patients diagnosed with OSA. **(A)** NAFLD activity score. **(B)** *CD36* mRNA levels. **(C)** Correlation in the study population of matched mRNA values for CD36 with the indicated respiratory parameters, evaluated by Spearman's *r*-test. **(D)** Representative 20X and 60X images of CD36 immunostaining, and quantification of CD36-expressing cells. Scale bar 100 and 50 μm, respectively. Study population: control group (No-OSA) (*n* = 11) and OSA patients (*n* = 9). *p < 0.05, OSA vs. Control, compared using the Mann-Whitney *U*-test.

Although no significant differences were observed in liver enzymes between the two groups, there was a higher prevalence of NAFLD and evidence of more severe disease among patients with OSA (Table 1 and Figure 1A). Surprisingly, 36.4% of patients without OSA exhibited NAFLD, all of them featuring simple steatosis. In the OSA group, however, 55.6% of them had simple steatosis and 22.2% showed histological features of steatohepatitis (NASH) (Table 1 and Figure 1A). Hepatic fibrosis was not observed. All other variables did not significantly differ between groups.

### Expression of CD36 Is Increased Within the Liver of OSA Patients

Next, we wanted to investigate whether OSA might alter CD36 expression in the liver. Interestingly, hepatic mRNA levels of CD36 were significantly higher in patients with OSA when compared with control patients (Figure 1B), and its expression significant positively correlated with both AHI and ODI values in the entire study population (Figure 1C), but not with Tc90 (Supplementary Figure 1A). In parallel, an increase of CD36 protein expression was also observed in the livers from OSA patients compared with those from the control group detected by immunostaining (Figure 1D).

### Intermittent Hypoxia (IH) Triggers Hepatic Steatosis in Mice

**Figure 2:**
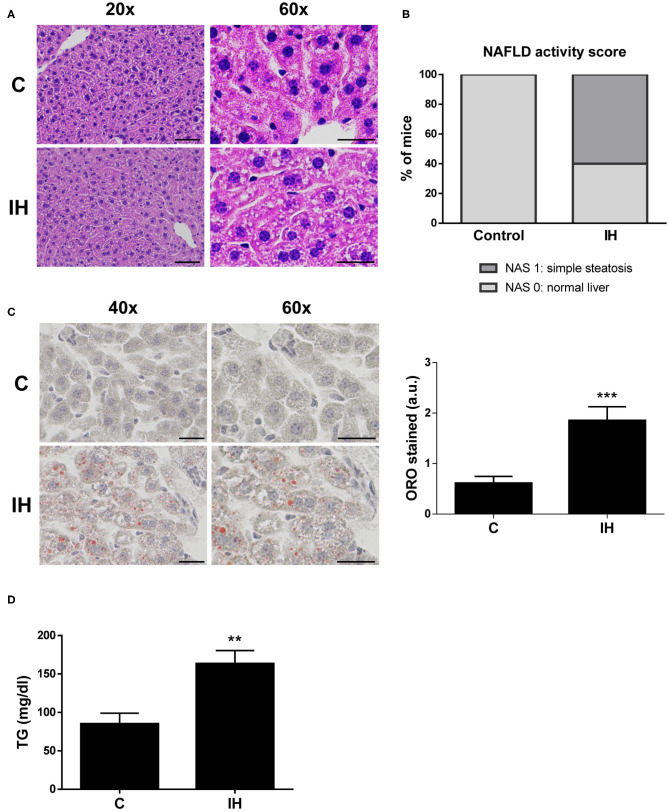
Intermittent Hypoxia (IH) is associated with increased hepatic lipid content in mice. **(A)** Representative 20X and 60X images of liver sections stained with hematoxylin and eosin (H&E). Scale bar 100 and 50 μm, respectively. **(B)** NAFLD activity score. **(C)** *(left panel)* Representative 40X and 60X images of Oil Red O stained liver sections. Scale bar 50 μm. *(right panel)* Quantification of red-stain intensity. **(D)** Analysis of hepatic triglyceride (TG) levels. Experimental groups: mice maintained in normoxic conditions (Control, C) and mice exposed to intermittent hypoxia (IH) (*n* = 10 mice in each group). **p < 0.01 and ***p < 0.005, IH vs. C, compared using the unpaired *t*-test.

In order to investigate whether intermittent hypoxia (IH), one of the main features of OSA, contributes to liver steatosis and to the increase of CD36 expression observed in OSA patients, a mouse experimental model of OSA was used. After 2 months of IH exposure, histological examinations of liver tissue revealed that 60% of the mice submitted to IH exhibited signs of mild hepatic steatosis while control mice displayed normal liver features (Figures 2A,B). Liver fibrosis was not detected in any of the groups (Supplementary Figure 2).

Next, we investigated the amount of lipids performing an Oil Red O staining on sections of liver biopsy samples from all mice. The results indicated that there was a significant increase in average red-stain intensity (directly proportional to lipid content) among the IH mice when compared to control mice (Figure 2C). To further evaluate hepatic lipid content, triglycerides were extracted and quantified from liver biopsies of both IH and C mice. Triglyceride levels of the IH mice were greater than those observed in the C mice (Figure 2D).

### Intermittent Hypoxia (IH) Induces Hepatic CD36 Expression

**Figure 3:**
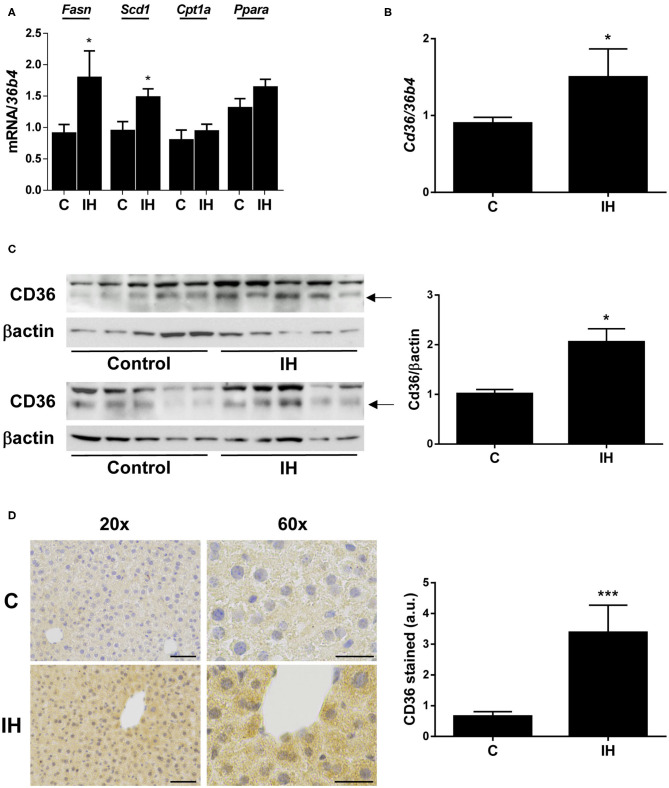
Intermittent Hypoxia (IH) induces hepatic CD36 expression in mice. **(A)** mRNA levels of *Fasn, Scd1, Cpt1a*, and *Ppara*. **(B)** *Cd36* mRNA levels. **(C)** *(left panel)* Representative blots with the indicated antibodies. *(right panel)* Quantification of all blots with respect to loading control, βactin. **(D)** *(left panel)* Representative 20X and 60X images from CD36 immunochemistry. Scale bar 100 and 50 μm, respectively. *(right panel)* Quantification of CD36-stain intensity. Experimental groups: mice maintained in normoxic conditions (Control, C) and mice exposed to intermittent hypoxia (IH) (*n* = 10 mice in each group). *p < 0.05 and ***p < 0.005, IH vs. C, compared using the unpaired *t*-test.

tHEN, we analyzed the hepatic expression of genes involved in the regulation of lipid metabolism. We observed a significant increase in the expression of genes implicated in lipid synthesis, such as *Fasn* (fatty acid synthase) and *Scd1* (stearoyl-CoA desaturase 1), among livers from mice exposed to IH (Figure 3A); however, no differences were observed in the expression of genes implicated in β-oxidation, such as *Cpt1a* (carnitine palmitoyltransferase I) and *Ppara* (peroxisome proliferator activated receptor alpha) (Figure 3A).

With respect to CD36, its hepatic mRNA expression was significantly increased in mice submitted to IH compared to those maintained in normoxic conditions (Figure 3B), which was also found in OSA patients. In parallel, its protein expression determined by both Western blot (Figure 3C) and immunohistochemistry (Figure 3D) was elevated in the livers of IH mice.

## Discussion

Distinct clinical studies have reported that OSA is significantly associated with NAFLD severity (26) and there is an increasing experimental evidence that chronic IH, the best characterized OSA manifestation, is a major trigger for oxidative stress and inflammatory liver injury leading to NAFLD progression (17). In agreement with these previous studies, we found that 22.2% of OSA patients had NASH whereas none of those without OSA had histological features of NASH, supporting the assumption that OSA is a risk factor for progression from simple steatosis to NASH (27). Interestingly, our findings showed that there were no differences regarding BMI between the two study groups, but waist circumference was significantly higher in patients with OSA, suggesting that is abdominal obesity, but not overall obesity, what actually has a clinical impact on the features of metabolic syndrome, including OSA and NAFLD.

To the best of our knowledge, this is the first study revealing that CD36 expression is significantly elevated in livers from patients with OSA. Moreover, both AHI and ODI positively correlated with hepatic *CD36* mRNA levels, indicating a potential role for nocturnal IH in the upregulation of this FFA transporter. An intriguing question regarding our findings showed herein is whether age might influence the hepatic CD36 expression pattern observed in OSA patients because they were significantly older (54.6 ± 10.6 years) than those without OSA (39.9 ± 9.6 years). Indeed, we have reported that hepatic CD36 expression increased with aging in mice and humans (13), but the age-dependent increases in hepatic CD36 expression were observed comparing young (20–38 years old) with aged individuals (50–83 years), thus we believe that age differences seen in our study population are not sufficient to explain the hepatic CD36 upregulation observed in OSA patients. Supporting this assumption, no correlation was found between *CD36* mRNA expression and age in our study population (Supplementary Figure 1B).

In agreement with our findings in OSA patients, the majority of mice exposed to IH displayed simple steatosis as well as a higher hepatic TG content and CD36 expression than in control mice breathing normal oxygen concentrations. Collectively, our results indicate that IH may contribute to hepatosteatosis setup, partly by the upregulation of hepatic CD36 expression, but the underlying molecular mechanisms still remain to be elucidated.

It is well-known that cellular adaptive responses to hypoxia are tightly regulated by hypoxia-inducible transcription factors (HIFs), being HIF1α and HIF2α the best characterized (28). In that regard, Li et al. demonstrated that IH exacerbated hepatosteatosis in mice in parallel with an upregulation of key genes for hepatic lipid biosynthesis, such as *Srebp1* and *Scd1* (20) and that this effect is mediated through HIF1α (29). In line with these results, our study also found increased mRNA levels of relevant genes for *de novo* lipogenesis, such as *Fasn* and *Scd1*, along with an upregulation of *Cd36* gene expression in livers of mice exposed to IH, suggesting that HIF1α might regulate *Cd36* gene expression as well. There is convincing evidence, however, indicating that the regulation of CD36 expression is not largely linked to the HIF1α/SREBP1c signaling pathway in hepatocytes. Notably, it has been recently reported that hepatocyte-specific *Srebp1* downregulation did not affect expression of genes involved in FFA uptake as *Cd36* in mouse livers (30). In addition, we have just demonstrated that both CD36 expression and triglyceride content increased in mouse and human liver cells under hypoxic conditions and that silencing *HIF2A* gene markedly suppressed both *CD36* gene upregulation and lipid accumulation in hepatocytes (14). The novelty of our present study is that both CD36 expression and the degree of steatosis are increased in livers from animal models of IH and in patients with OSA featured by nocturnal IH, supporting the notion that CD36 could be a key factor driving hepatosteatosis in OSA patients.

In conclusion, the results of the present study demonstrate that CD36 expression is increased within the liver of patients with OSA and in mice exposed to IH, the clinical hallmark featuring OSA. Moreover, our results point out that the excessive lipid accumulation observed in livers of mice under IH conditions is likely due to the upregulation of CD36, which is involved in FFA uptake into hepatocytes, along with that of genes implicated in *de novo* lipogenesis, thus leading to the onset of hepatosteatosis, the earliest phase of NAFLD. Collectively, our findings shed light on the molecular mechanisms underlying IH-induced hepatosteatosis helping to understand better the NAFLD pathogenesis and identifying CD36 as a potential target for new pharmacological therapies to NAFLD patients.

## Data Availability Statement

All datasets presented in this study are included in the article/Supplementary Material.

## Ethics Statement

The studies involving human participants were reviewed and approved by Human Ethics Committee of the Hospital Universitario Santa Cristina. The patients/participants provided their written informed consent to participate in this study. The animal study was reviewed and approved by Ethical Committee of the University of Barcelona.

## Author Contributions

CG-M, IA, and ÁG-R designed the study. EP-M, PL, and CG-M carried out and analyzed the clinical study. ER, BB, PM, and SI carried out the experimental study. RF, CG-M, IA, and ÁG-R analyzed and discussed data. IA and ÁG-R wrote the manuscript. All authors were involved in editing the paper and had final approval of the submitted and published versions.

## Conflict of Interest

The authors declare that the research was conducted in the absence of any commercial or financial relationships that could be construed as a potential conflict of interest.

## Supplementary Material

Supplementary Table 1 Primer sequences for RT-qPCR.

Supplementary Figure 1 Correlation in the study population of matched mRNA values for CD36 with Tc90 values **(A)** and age **(B)**, evaluated by Spearman's *r*-test. Study population: control group (No-OSA) (*n* = 11) and OSA patients (*n* = 9).

Supplementary Figure 2 Representative images of liver sections stained with Masson's trichrome solution. Experimental groups: mice raised in normoxic conditions (Control, C) and mice exposed to intermittent hypoxia (IH) (*n* = 10 mice in each group).
