# Supplementary material for: Intrahepatic Expression of Fatty Acid Translocase CD36 Is Increased in Obstructive Sleep Apnea
Source: Front Med (Lausanne). 2020 Aug 11;7:450. doi: 10.3389/fmed.2020.00450 (PMC7431763; doi:10.3389/fmed.2020.00450)
Supplement: Supplementary Table 1 — Primer sequences for RT-qPCR. [file Table_1.DOCX]

***Supplementary Table 1. Primer sequences for RT-qPCR.***

| **Gene** | **Forward (5’**🡪**3’)** | | **Reverse (5’**🡪**3’)** |
| --- | --- | --- | --- |
| m-*Cd36* | | AGATGACGTGGCAAAGAACAG | CCTTGGCTAGATAACGAACTCTG |
| m-*Cpt1a* | | TCAATCGGACCCTAGACACC | CTTTCGACCCGAGAAGACCT |
| m-*Fasn* | | CCCTTGATGAAGAGGGATCA | ACTCCACAGGTGGGAACAAG |
| m-*Ppara* | | AGAGCCCCATCTGTCCTCTC | ACTGGTAGTCTGCAAAACCAAA |
| m-*Scd1* | | TTCTTGCGATACACTCTGGTGC | CGGGATTGAATGTTCTTGTCGT |
| m-*36b4* | | AGATGCAGCAGATCCGCAT | GTTCTTGCCATCAGCACC |
| h-*CD36* | | ATGTGTGTGGAGAGCGTCAACC | TGAGCAGAGTCTTCAGAGACAGCC |
| h-*36B4* | | CAGGCGTCCTCGTGGAAGTGAC | CCAGGTCGCCCTGTCTTCCCT |
|  | |  |  |
