# Supplementary figures and images for: Intrahepatic Expression of Fatty Acid Translocase CD36 Is Increased in Obstructive Sleep Apnea
Source: Front Med (Lausanne). 2020 Aug 11;7:450. doi: 10.3389/fmed.2020.00450 (PMC7431763; doi:10.3389/fmed.2020.00450)

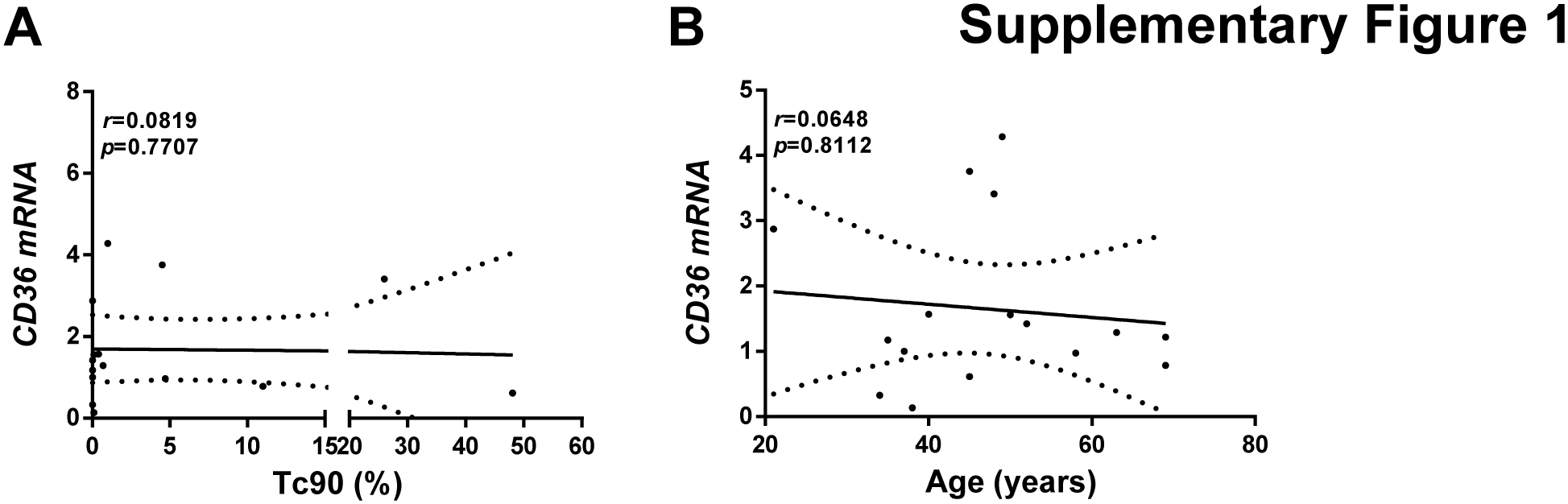

Supplement: Supplementary Figure 1 — Correlation in the study population of matched mRNA values for CD36 with Tc90 values (A) and age (B), evaluated by Spearman's r-test. Study population: control group (No-OSA) (n = 11) and OSA patients (n = 9). [file Image_1.TIF]

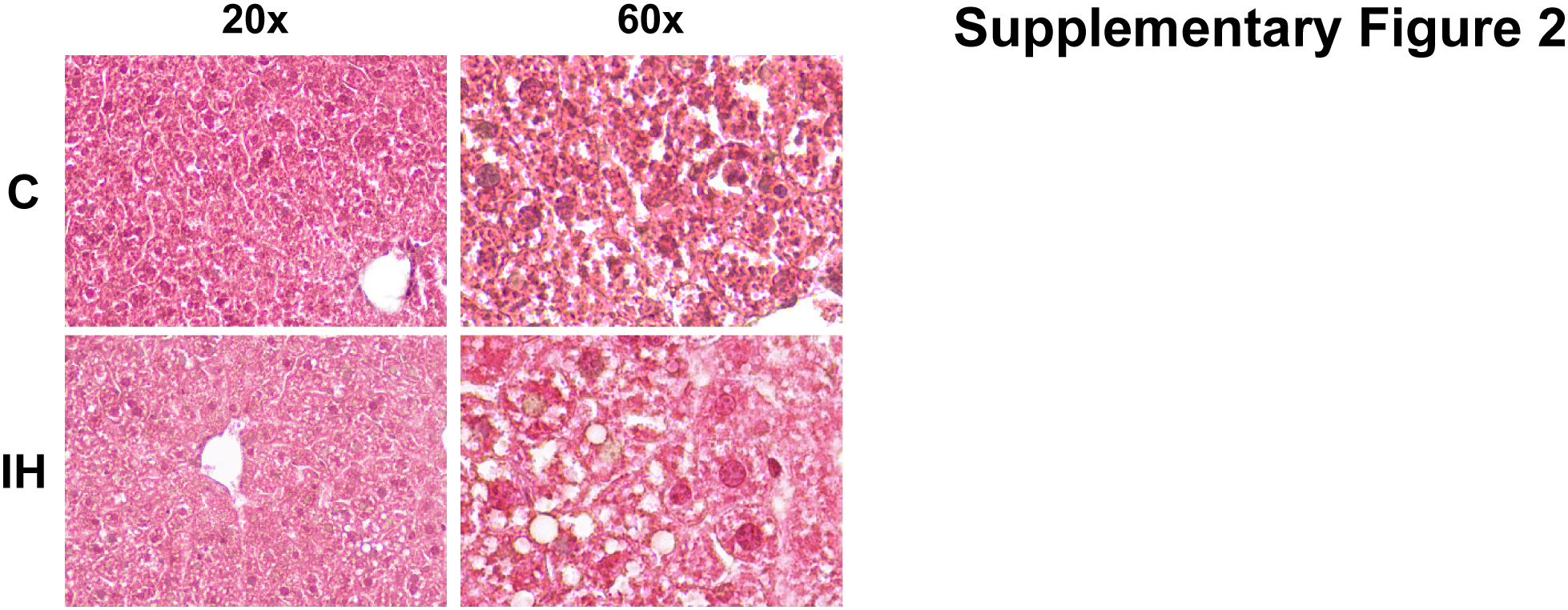

Supplement: Supplementary Figure 2 — Representative images of liver sections stained with Masson's trichrome solution. Experimental groups: mice raised in normoxic conditions (Control, C) and mice exposed to intermittent hypoxia (IH) (n = 10 mice in each group). [file Image_2.TIF]
